# Neutrophil elastase in the development of nephrogenic systemic fibrosis (NSF)-like skin lesion in renal failure mouse model

**DOI:** 10.1371/journal.pone.0259211

**Published:** 2021-10-27

**Authors:** A. Adhipatria P. Kartamihardja, Syahla Nisaa Amalia, Akiko Sekiguchi, Anu Bhattarai, Ayako Taketomi-Takahashi, Sei-ichiro Motegi, Hiroshi Koyama, Yoshito Tsushima

**Affiliations:** 1 Department of Diagnostic Radiology and Nuclear Medicine Department, Gunma University Graduate School of Medicine, Maebashi, Japan; 2 Department of Nuclear Medicine and Molecular Imaging, Universitas Padjajaran, Sumedang, Indonesia; 3 Department of Dermatology, Gunma University Graduate School of Medicine, Maebashi, Japan; 4 National Academy of Medical Sciences (NAMS), Bir Hospital, Kathmandu, Nepal; 5 Department of Public Health, Gunma University Graduate School of Medicine, Maebashi, Japan; 6 Division of Integrated Oncology Research, Gunma Initiative for Advanced Research, Gunma University Graduate School of Medicine, Maebashi, Japan; Wayne State University, UNITED STATES

## Abstract

Although neutrophil elastase (NE) may play a role in lung fibrosis and liver fibrosis, NE involvement in the development of nephrogenic systemic fibrosis has been unclear. We investigated the involvement of NE in the development of nephrogenic systemic fibrosis-like skin lesions post-injections of linear gadolinium-based contrast agents in renal failure mouse models. Renal failure mouse models were randomly divided into three groups: control group (saline), gadodiamide group, and gadopentetate group. Each solution was intravenously administered three times per week for three weeks. The mice were observed daily for skin lesions. Quantification of skin lesions, infiltrating inflammatory cells, and profibrotic cytokines in the affected skin was performed by immunostaining and reverse-transcription polymerase chain reaction (RT-PCR). Blood samples were collected from the facial vein to quantify NE enzymatic activity. The ^158^Gd concentrations in each sample were quantified using inductively coupled plasma mass spectrometry (ICP-MS). In the gadodiamide group, the mRNA expression of fibrotic markers was increased in the skin lesions compared to the control group. In the gadopentetate group, only collagen 1α and TGF-β mRNA expression were higher than in the control group. The expression of CD3+, CD68+, NE cells and the NE activity in the blood serum were significantly higher in the gadodiamide and gadopentetate groups compared to the control group. Gadolinium concentration in the skin of the gadodiamide group was significantly higher than the gadopentetate group, while almost no traces of gadolinium were found in the control group. Although gadopentetate and gadodiamide affected the fibrotic markers in the skin differently, NE may be involved in the development of fibrosis linked to the GBCAs injections in renal failure mouse models.

## Introduction

Nephrogenic systemic fibrosis (NSF) is a disease found in patients with acute or chronic renal impairment, especially those with end-stage renal disease (ESRD). NSF has been highly linked with the exposure of gadolinium (Gd)-based contrast agents (GBCAs), in particular linear-chelate GBCAs [1, 2]. A population-based study on patients with ESRD showed that each contrast-enhanced MRI (using Gd) presented a 2.4% risk for NSF [3]. The symptoms may clinically present as symmetrical skin thickening beginning at the limbs [1, 4]. Evidently, traces of Gd were found in the skin and soft tissue of patients who suffered from NSF [5]. Animal studies also confirmed that the Gd from linear GBCAs were retained in various organs at a much higher concentration than from macrocyclic GBCAs [6, 7], and were detected in several molecular sizes [8]. Gd retention was increased in many organs of the renal failure mouse model [6].

Although the incidence of NSF has been kept at bay with extra precaution when patients have renal impairment [9], the definitive pathological mechanism has yet to be explained. Histologically, NSF has similarities with scleroderma, which is characterized by progressive scleromyxedema-like fibrosis of many organs, including skin, muscle and lungs [10]. Biopsied lesions of scleroderma patients have increased neutrophils compared with normal tissue. In the pathologic process of inflammatory disease such as fibrotic disorders, neutrophil elastase (NE) released by neutrophils may be involved in a stimulatory activity for collagen synthesis in fibroblasts [11, 12]. Elevation of NE has also been reported in patients with systemic sclerosis, which may be associated with lung fibrosis [12]. NE has also been confirmed to have a role in fibrotic disorders in a study showing that elastase-deficient mice were resistant to bleomycin-induced fibrosis, and that treatment with elastase inhibitors abolished fibrosis in this model [13]. The activation of neutrophils in patients with CKD may lead to oxidative stress and inflammation, which may worsen with the involvement of pro-inflammatory cytokines [14]. To the best of our knowledge, there has not been a report about the NE activity in patients with NSF. Gd has been associated with stimulation of fibroblasts [15–17]. Fibroblast specimens collected from patients with NSF showed markedly increased production of collagen, fibronectin and hyaluronic acid production [18]. Thus, Gd may be involved in the process of fibrosis in renally impaired patients, which may be related to NE. The method to produce renal failure mouse model has been established by bilateral electrocoagulation of the renal cortex. The thermal damage to renal tissue by electrocoagulation induced tissue necrosis and renal atrophy, resulting in uremic that leads to stable renal failure [19].

In this study, we investigated the NE activity in a chronic renal failure mouse model after a series of injections of two types of linear-chelate GBCAs, along with the histological changes in the mice’s skin.

## Materials and methods

### Animals

All procedures in this study were performed under the guidelines that have been approved by the Institute and Committee for Animal Use and Care of Gunma University made in accordance to the ARRIVE guidelines 2.0 to minimize animal suffering and reduce the number of animals used [20]. Animal were housed in cages (5 mice per L-sized cage) under a 12-h dark/light cycle, with food and water *ad libitum*. Twenty-five female ddy mice (five weeks old; weight 25.7 ± 0.7 g [mean ± SD]) were used in this study. Electrocoagulation was performed on both kidneys as described by Gagnon et al. [19] to create a renal failure model. In brief, the procedure was performed under general anesthesia by 3% inhaled isoflurane supplemented with 2 mL/min of air. The mice were placed on the opposite side of the incision site on a heating pad covered with a clean paper towel. After a 2-cm incision was made, the entire kidney was carefully exteriorized. Electrocoagulation was performed on the kidney surface excluding the hilum approximately 1 mm deep and 2 mm apart. Then, the kidney was returned to the retroperitoneal cavity, and the wound was sutured closed. The other kidney underwent similar surgery after a week-long recovery period. Renal failure was confirmed by measuring serum creatinine with a commercially available creatinine assay kit (Crystal Chem Inc., IL) 12 weeks post-surgery. The creatinine level of the mice was 2.63 ± 0.1 mg/dL at the start of the study. These renal-failure mice were randomly divided into three groups to be injected with either gadodiamide (n = 10), gadopentetate (n = 10) or saline (n = 5).

### GBCAs injection protocol

Omniscan (gadodiamide, 0.5 mol/L; Daiichi-Sankyo Co. Ltd., Tokyo, Japan) and Magnevist (gadopentetate, 0.5 mol/L; Bayer Yakuhin Ltd., Osaka, Japan) were purchased from each respective company. Each agent was intravenously administered via the tail vein every other day (total = nine injections for three weeks at a dose of 1.8 mmol/kg (equivalent to 0.15 mmol/kg in human [21]). Mice in the control group received 200μL saline in a pattern similar to the treated group. The mice were observed daily, and the samples were collected at two weeks after the first development of skin lesions.

### Neutrophil elastase (NE) enzymatic analysis

The enzymatic analysis of NE activity was performed using Mouse Neutrophil Elastase ELISA Kit (ab 252356, Abcam plc., Cambridge, MA). In brief, the mouse’s blood was collected in a tube from the facial vein. After clot formation, the blood was centrifuged at 2000G for 10 min to collect the serum. The serum for analysis was carefully prepared according to the kit protocol. Finally, a microplate reader (Bio-Rad 680, Bio-Rad Laboratories Inc., CA) was used to measure the colorimetric endpoint at OD 450 nm. The NE concentration in the sample was calculated by interpolating the blank-subtracted absorbance values against the standard curve (pg/mL).

### Histochemical and immunohistochemical analysis

The mice were sacrificed by deep anesthesia using 5% of isoflurane (performed by inhalation), followed by cervical dislocation. The skin from the area with the lesion was removed, fixed with formaldehyde, and embedded in paraffin. In mice without skin lesions, skin from similar areas was selected. 3 μm sections were stained with hematoxylin (H&E) and eosin or Masson Trichome. A measurement of dermal thickness, defined as the distance from the epidermal-dermal junction to dermal-subcutaneous junction, was performed in six random microscopic fields to assess skin fibrosis. For immunochemical staining, deparaffinized sections were boiled for antigen retrieval with a pressure cooker for 10 minutes and treated with endogenous peroxidase blocking reagent (DAKO, Glostrup, Denmark) for five minutes followed by 10 minutes of protein block (DAKO, Glostrup, Denmark) at room temperature. The sections were then incubated with the designated antibodies (Abs) overnight at 4°C, followed by incubation with horseradish peroxidase-labeled polymer-conjugated secondary Abs (ENVISION: DAKO, Glostrup, Denmark). The immunoreactivity was visualized using 3,3’-diaminobenzidine tetra-hydrochloride followed by counterstain with Mayer’s hematoxylin. Samples were evaluated by counting the number of positive cells, then calculating the average number present in serial sections. The dermal thickness was measured from H&E-stained sections using ImageJ software (version 1.8.0. NIH, Bethesda, MD).

Abs and their sources were: rabbit anti-mouse CD3 polyclonal Ab (pAb) (ab5690; Abcam plc., Cambridge, MA), mouse anti-mouse Neutrophil Elastase mAb (sc-55549, Santa Cruz Biotech, Inc., Dallas, TX), and rat anti-mouse CD68 mAb (MCA1957GA, Bio-Rad Laboratories Inc., CA). HRP-conjugated goat anti-mouse or anti-rabbit secondary Abs were obtained from Dako (Glostrup, Denmark).

### Reverse transcription-polymerase chain reaction (RT-PCR)

Total RNA was isolated using RNeasy Mini Kits (Qiagen, Valencia, CA), and subjected to reverse transcription using GoScript™ Reverse Transcription System for RT-PCR (Promega) according to the manufacturer’s instructions. Quantitative RT-PCR (qRT-PCR) was performed with the SYBR system (Applied Biosystems, Foster City, CA), using ABI 7300 real-time PCR instrumentation (Life Technologies). SYBR probes and primers for collagen 1α, CTFG, αSMA, Hyal-1, Hyal-2, HAS-1, HAS-2, HAS-3, TGF-β, IL-6, and Glyceraldehyde-3-phosphate dehydrogenase (GAPDH) were purchased from Sigma (St. Louis, MO) and Takara Bio Inc. (Otsu, Japan). The levels of GAPDH were quantified in parallel with the target genes as an internal control. Normalization and fold-changes were calculated using the comparative Ct method.

### Retained Gd analysis by inductive coupled plasma mass spectrometry (ICP-MS)

Each sample was weighed to be digested with 600 μL of nitric acid (HNO_3_) and 200 μL of hydrogen peroxide (H_2_O_2_) in a perfluoroalkoxy vial. After digestion with eight-sequences of microwave program for 125 min (mls 1200 mega, Milestone Inc. Shelton, CT), ultra-purified MQ water was added to each sample for a total volume of 10 mL. The Gd isotope (^158^Gd) in each sample was measured by inductively coupled plasma-mass spectrometry (ICP-MS) using the ELAN® DRC II instrument (PerkinElmer, Inc. Waltham, MA).

#### Statistical analysis

The analysis between the groups were determined by Student’s *t*-test or analysis of variance followed by *post-hoc* Tukey’s honest significant difference (HSD) test with SPSS software (version 23; IBM-SPSS, Inc., Chicago, IL) and were shown in graphs as mean ± standard error margin (SEM). A *p* value < 0.05 was considered statistically significant.

## Results

### Development of skin lesions after the GBCAs injection

No skin lesions were observed in the control group. In the gadodiamide group, seven of ten mice developed skin lesions in different places, while three showed no visible skin lesions. One mouse showed an 8.3 x 6.2 mm indurated erosion under the muzzle, an erythematous nodule with hair loss behind the right ear (5.1 mm x 3.6 mm), and an erythematous macule with hair loss on the shoulder. Hair loss without skin involvement was also observed on the head, shoulder, between the eyes, and muzzle area.

In the gadopentetate group, eight mice suffered from different degrees of hair loss without skin erythema located symmetrically on the muzzle. One mouse showed a visible skin lesion on the dorsal neck (9.4 mm x 5.1 mm) while the other showed a lesion on the top of the head (3.6 mm x 3.4 mm) and between the eyes.

### Fibrotic markers in the skin lesion (Fig 1)

**Fig 1:**
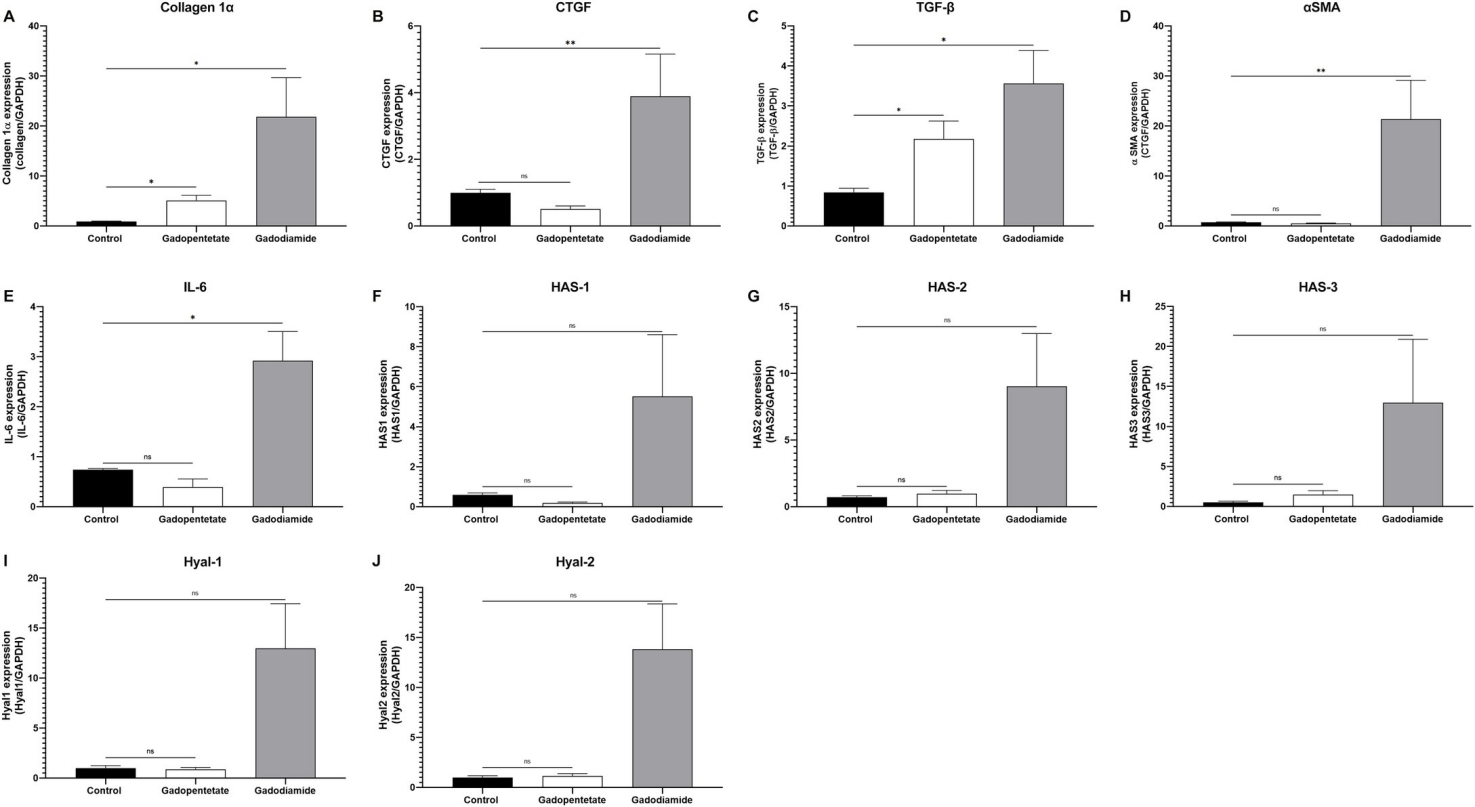
The mRNA expression of the fibrotic marker in the mice skin with lesion. mRNA levels of Collagen 1α, CTGF, αSMA, TGF-β, HAS-1, HAS-2, HAS-2, HAS-3, Hyal-1, and Hyal-2 were evaluated in the skin lesion after Gd injection. Mice with severe skin lesions in the gadodiamide group showed high fibrotic markers similar to those found in NSF skin lesions (A-E, G, I-J). In mice with mild lesions, HAS-1 and HAS-3 were not increased while other mRNA levels were (F, H). In the gadopentetate group, collagen 1α and TGF-β mRNA expression were increased, while CTGF expression was decreased (A-C). Other mRNA expression in the gadopentetate group were similar to the control group. mRNA level in control mice were assigned as values of 1. ns = not significant, *p < 0.05, **p < 0.01.

The mRNA expression of collagen 1α, CTGF, TGF-β, αSMA, and IL-6was significantly higher in the gadodiamide group compared to the control group (*p* < 0.01, Fig 1). Expression of collagen 1α and TGF-β mRNA in the gadopentetate group was significantly higher than that in the control group (*p* < 0.05), while the expression of other markers was similar to that of the control group.

### Histopathological analysis in the skin lesion

**Fig 2:**
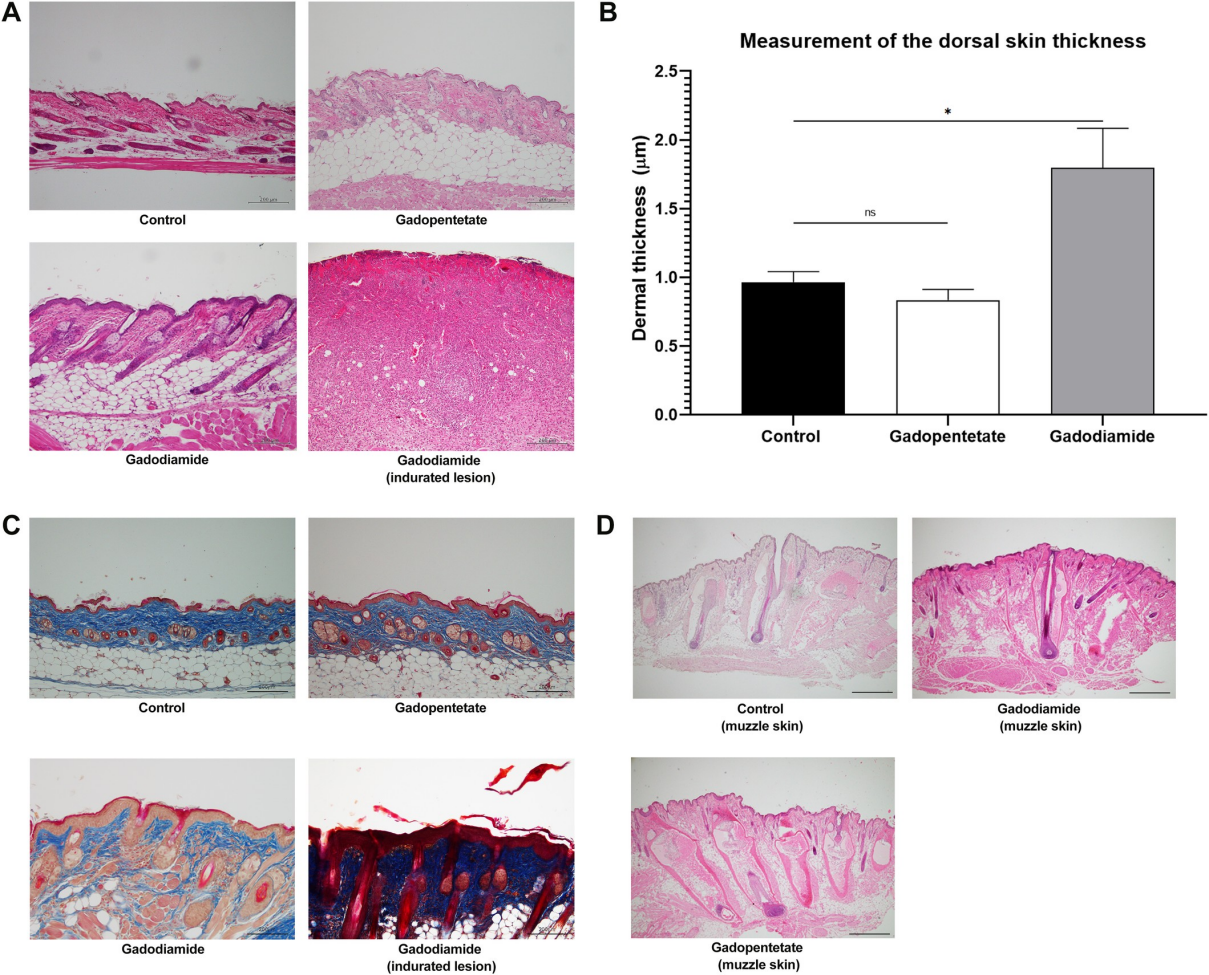
Skin alteration after gadolinium injection. Representative photomicrographs of haematoxylin and eosin staining (A) or Masson’s trichrome staining (C) show effects of different GBCA injections on the skin. Scale bar = 200 μm. Quantification of epidermal thickness in mice after receiving gadolinium injection (B). The dermal thickness in the gadopentetate group and gadodiamide group with mild lesions were similar to the control group, while mice with severe lesion had thicker dermis. Representative photomicrographs of haematoxylin and eosin staining (D) taken from the muzzle area show destroyed dermal papilla after the injection of gadopentetate. Picture on the lower right (D) is a 10x magnification of gadopentetate-injected muzzle skin. Scale bar = 200 μm. All data represent the mean score ± SEM. ns = not significant **p < 0.01.

Histopathological examination by H&E staining revealed that mice in the gadodiamide group had the greatest dermal thickness (Fig 2A). The injection of gadodiamide significantly increased the dermal thickness compared to the control (*p* < 0.05) and gadopentetate groups (*p* < 0.05). The dermal thickness of mice in the gadopentetate group was also similar to the control group (Fig 2B). We also confirmed increased collagen deposition by Masson’s trichrome staining in the gadodiamide group compared to the control and gadopentetate groups (Fig 2C).

**Fig 3:**
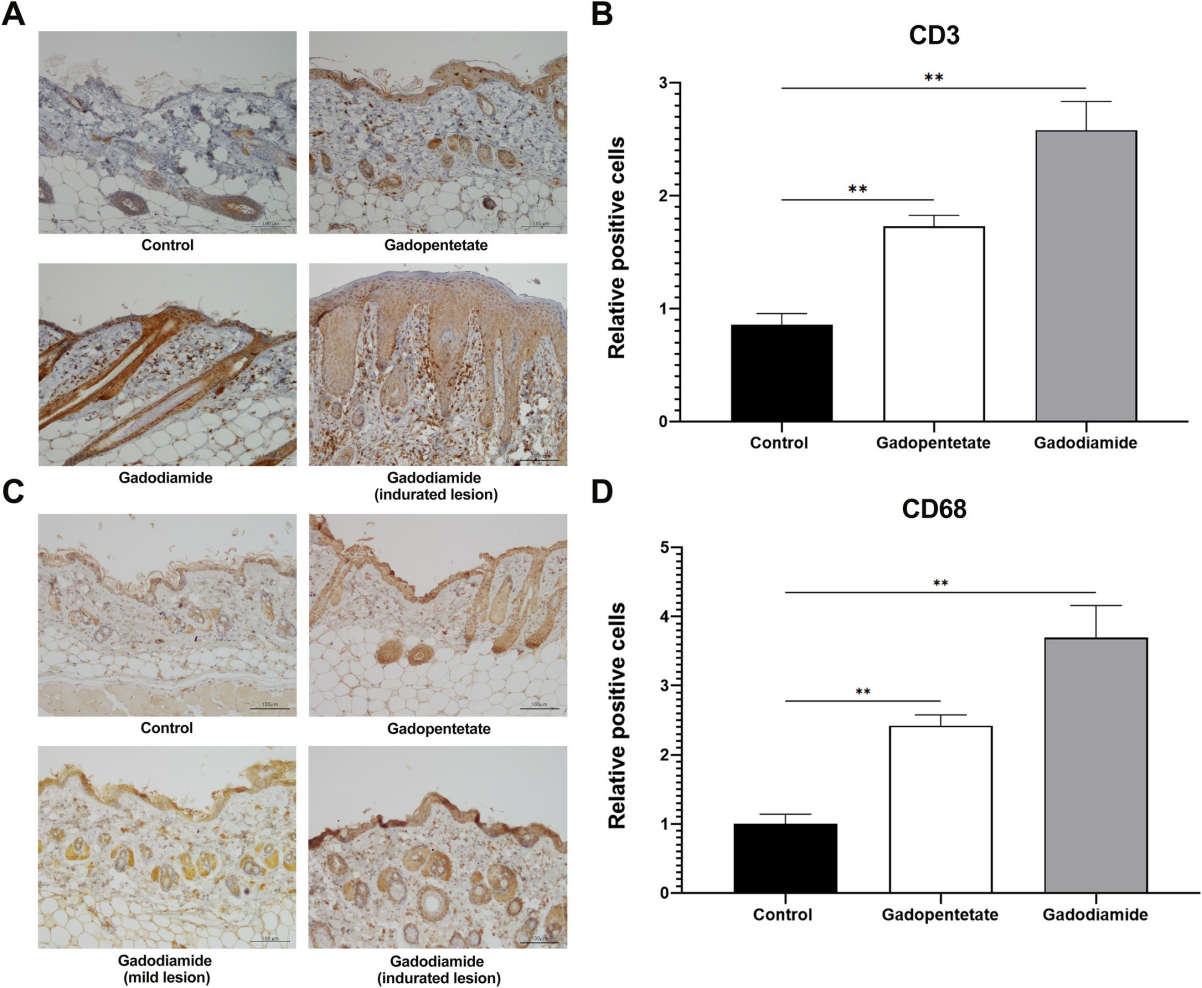
Effect of GBCAs injection on the CD3 and CD68 expression on the skin. Representative photomicrographs of CD3^+^ T cells (A) and CD68^+^ macrophage immunostaining (C). Quantification number of infiltrated CD3^+^ T cells (B) and CD68^+^ macrophages (D) in the dermis, determined by counting cells in six random microscopic fields. The expression of CD3^+^ cells was significantly higher in the gadodiamide group compared to the control group, in mice with mild lesions and mice with severe lesions. Similar results were also observed in the CD68^+^ expressed cells. Scale bar = 100 μm. The expression of positive cells in control mice was assigned values of 1. All data represent the mean score ± SEM. **p < 0.01.

Gd injection caused hair loss in eight mice, especially in the muzzle area of gadopentetate-treated mice. In the muzzle area, the hair follicles in the gadopentetate group showed damaged dermal papilla, which may play a crucial role in hair growth. Hair follicles of the gadodiamide and control groups looked intact (Fig 2D). Gadodiamide injection induced marked inflammatory cell infiltration with fibrosis in the skin. The numbers of infiltrated CD3^+^ T cells (Fig 3A, 3B) and CD68^+^ macrophages (Fig 3C and 3D) were significantly increased in the gadopentetate and gadodiamide group, rega3dless of skin lesion severity.

### The expression of NE and serum NE activity in the skin following GBCAs injection

**Fig 4:**
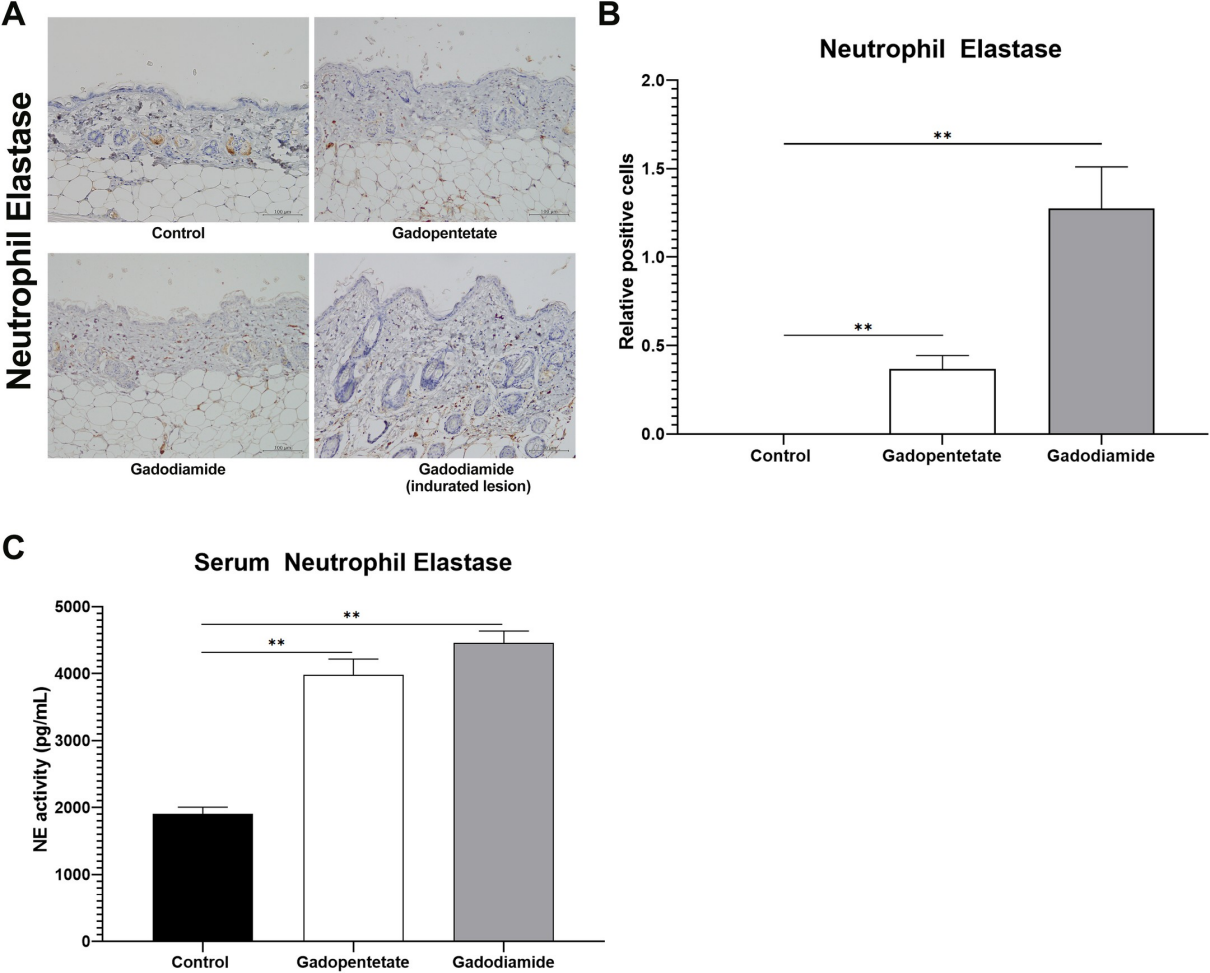
Effect of GBCAs injection on the production of Neutrophil Elastase (NE). Representative photomicrographs of NE immunostaining showing effects of different GBCA injections on the skin (A). Quantification number of NE expression in the dermis (B), determined by counting cells in six random microscopic fields. Injection of gadopentetate or gadodiamide increased the NE activity in the blood serum (C), and in the skin, regardless of the severity of skin lesions in both treated groups. Scale bar = 100 μm. The expression of positive cells in control mice was assigned values of 1. All data represent the mean score ± SEM. **p < 0.01.

The NE expression on the skin was strikingly higher in the gadodiamide group compared to the control group (*p* < 0.01), regardless of skin lesion severity (Fig 4B). The NE expression was also higher in the gadopentetate group compared to the control group (*p* < 0.01), while almost no expression of NE was observed in the skin of the control group (Fig 4A and 4B). Moreover, the NE activity measured from the blood serum showed that injection of either gadopentetate or gadodiamide significantly increased the NE activity (*p* < 0.01, Fig 4C).

### Gd concentration in the skin

**Fig 5:**
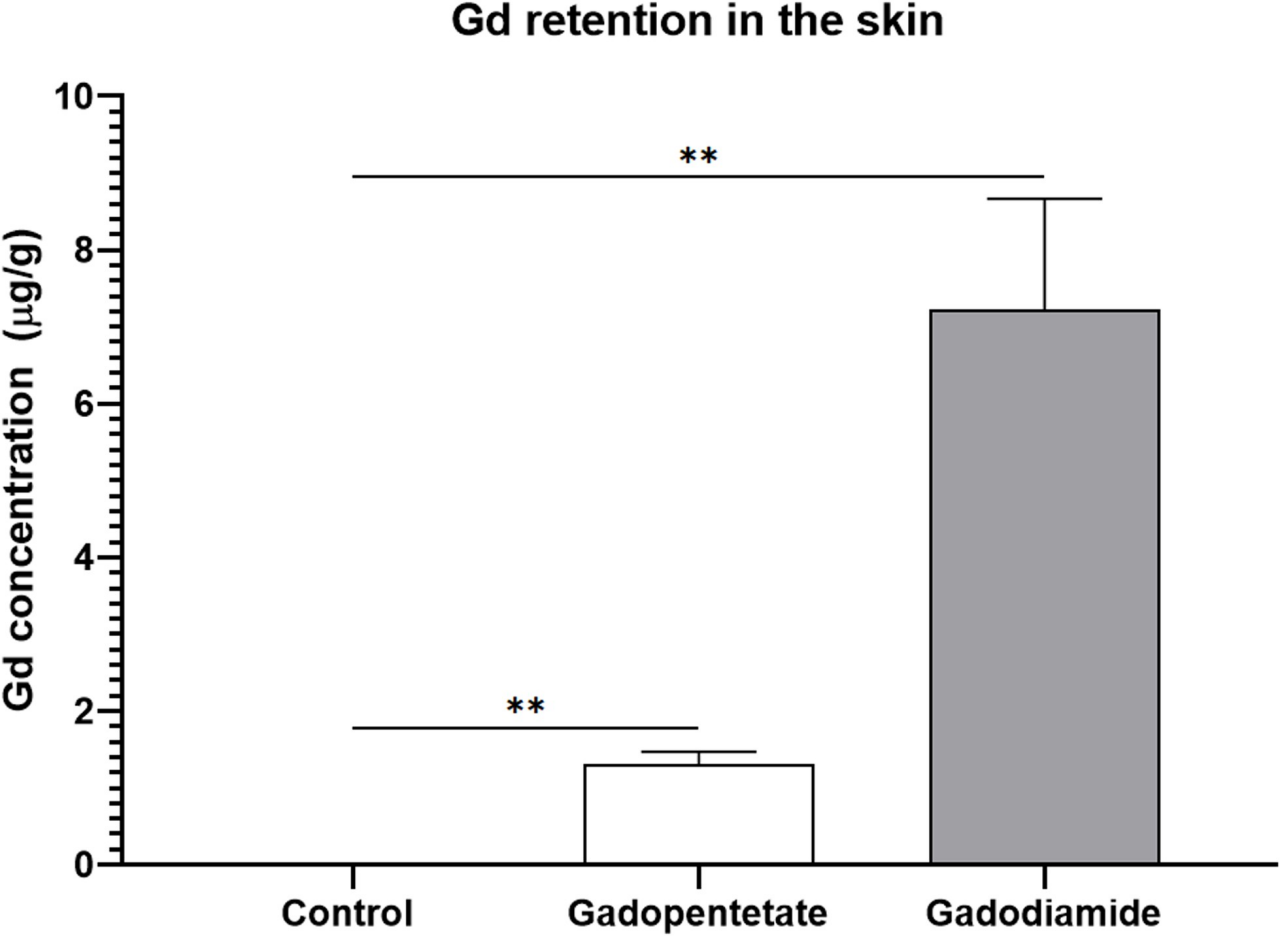
Gd retention in the skin of the renal failure mouse model after the injections of different GBCAs. Gd concentration of the gadodiamide group was significantly higher compared to the gadopentetate and control groups. In the gadodiamide group, Gd concentration in the mice with severe skin lesions was significantly higher than the Gd concentration in the mice with mild lesions. All data represented as the mean score ± SEM. **p < 0.01.

Quantification of ICP-MS experiment was calculated by a linear regression graph of standard Gd solution, and was verified in concentrations up to 300 μg/g. The limit of detection (LOD) and the limit of quantification (LOQ) were determined to be 0.0001 μg/g. In the gadodiamide group, the Gd retention in the skin (7.22 ± 5.2 μg/g) was significantly higher than the control group (0.004 ± 0.001 μg/g, *p* < 0.01). Gd retention in the gadopentetate group (1.31 ± 0.51 μg/g) was also significantly higher than the control group (*p* < 0.01, Fig 5). We found significant differences in Gd retention between the gadodiamide and gadopentetate groups (*p* < 0.01, Fig 5).

## Discussion

Injection of either gadodiamide or gadopentetate in the renal failure model led to the development of skin lesions, with more severe lesions in the gadodiamide group. Histologically, the skin lesions showed remarkably high lymphocyte infiltration and positive fibrotic markers, particularly in the gadodiamide group. The NE activity was higher in the gadodiamide or gadopentetate groups compared to the control group, suggesting the involvement of NE in the development of NSF-like skin fibrosis.

Typical early signs of NSF are symmetrical skin lesions in the extremities [22–24] and diffuse hair loss [22]. The gene expression in skin lesions of mice injected with gadodiamide showed a profile similar to that found in the skin of patients suffering from NSF. In our study, gadodiamide and gadopentetate induced infiltration of inflammatory cells into the skin. Despite almost all mice injected with gadopentetate developing symmetrical skin lesions on the muzzle, only the gene expression of collagen I and TGF-β were noticeably increased. Interestingly, CTGF expression was decreased compared to the control group. Although CTGF may be involved in TGF-β action in stimulating collagen production [25–27], the higher collagen expression in the gadopentetate group may be mostly stimulated by the increase of TGF- β rather than the CTGF. Wermuth et al. showed that although Gd exposure had no effect on the production of CTGF, the conditioned media taken from Gd-isolated peripheral blood monocytes successfully stimulated dermal fibroblasts to produce collagen *in vitro* [28]. A previous study showed that the semi-quantitative CTGF expression in patients with NSF skin lesions hardly showed any difference from the healthy control [29], indicating that CTGF may not be exclusively involved in the development of NSF-like skin lesions. The pathophysiology of the skin lesions from these two GBCAs may not be the same, even though they are both linear-chelate contrast agents.

Delayed elimination of GBCAs during renal impairment may increase GBCA de-chelation [30, 31]. Thus, the higher the dose, the higher the risk of developing NSF, due to the higher amount of free Gd released from the chelate. The Gd deposition in skin of animals with impaired renal function was found within collagen bundles and the fibroblast. Despite belonging to the linear group, Gd from gadodiamide was deposited significantly higher in the skin [32]. Gadodiamide has lower stability compared to gadopentetate [33], but gadodiamide was provided with the excess chelate that was supposed to prevent further transmetallation. However, the excess chelate may interfere with organic metals in the body. It has been reported that gadodiamide may cause a spurious decrease in serum calcium [34], and interfere total iron binding capacity [35]. This suggests that the excess chelate was not effectively prevent the transmetallation of gadodiamide, resulting in higher Gd concentration in the skin.

Several studies showed that GdCl_3_ administration may prevent fibrosis through the apoptosis pathway, resulting in the suppression of TGF- β 1 and αSMA [36].+ Isolated Kupffer cells exposed to GdCl_3_ may prevent liver fibrosis without affecting procollagen type I [37]. In addition, our previous study showed that although the injection of GdCl_3_ to the renal failure model might lead to the mouse’s demise, the remaining mice did not develop any skin lesions [6]. Therefore, it was difficult to conclude that the development of skin lesions in NSF was due to high concentrations of free Gd released from the chelate. The higher Gd retention found in the gadodiamide group may not necessarily be in the form of free Gd ions. In addition, both macrocyclic and linear GBCAs may increase the expression of types I and III collagen, fibronectin, and α-smooth muscle actin (α-SMA) in normal dermal fibroblasts [28]. Considering the co-expression of CD68 and α-SMA may contribute to the macrophage-to-myofibroblast transition [38, 39], especially during renal fibrosis [40], it may partially support the explanation that the more severe lesions in the gadodiamide group were because of the high expression of CD68 and α-SMA. High doses of gadopentetate or gadodiamide may also inhibit protein synthesis [18, 41], resulting in less stimulation of fibroblasts compared to lower doses [18]. Therefore, the possibility remains that chelated GBCA or Gd bound to endogenous macromolecules may contribute to the early development of NSF.

Previous studies regarding NE involvement in fibrosis was observed in lung fibrosis [42] and liver fibrosis [43], but whether such mechanisms were involved in the development of NSF-like skin lesion was still unclear. In our study, the increased expression of NE in the skin may be associated with the injection of either gadopentetate or gadodiamide, indicating the important role of NE in the development of NSF-like skin fibrosis after GBCA injection in the renal failure mouse model. Previous studies showed that NE was elevated in cases with inflammatory activity and fibrosis [44–46], thus NE may be beneficial as a serological marker for early detection of the NSF development in renal failure patients injected with GBCAs.

The NSF incidence rate was varied amongst GBCAs use in clinical settings with many confounding factors affecting the skin lesion development, such as GFR, iron level, metabolic condition, or chronic inflammation [47]. These factors may explain why few mice in our study did not develop skin lesions. Although not all mice developed fibrosis, the NE activity measured in the serum was uniformly increased in mice exposed to either gadopentetate or gadodiamide. Co-incubation of GdCl_3_ or GBCAs with lipopolysaccharide may increase the reactive oxygen species (ROS) production by macrophages, yet only linear GBCAs significantly affected the production of inflammatory cytokines [48]. GdCl_3_ may increase mesenchymal stem cell (MSC) proliferation and increase the expression of endothelin 1 (ET‐1) regulated not only by endothelial cells but also monocytes and macrophages [49]. ET-1 may be responsible for neutrophil activation and increase elastase activity [50], and has been implicated in the pathogenesis of skin fibrosis [51]. ET-1 production by human mesangial cells was affected by ROS [52], and was increased in chronic renal failure [53].

The transmetallation of Gd or the free chelate with endogenous molecules may be involved in NSF development by activating a series of complex mechanisms. The linear GBCAs, particularly gadodiamide, may stimulate the production of pro-inflammatory cytokines, such as TGF-β and IL-6, and stimulate the ROS expression by macrophages, increasing ET-1 production. Then, delayed elimination of GBCAs in renal failure may increase the event of transmetallation. Chronic renal failure and Gd from GBCAs may further increase the expression of ET-1. These processes may promote fibroblast activation that may lead to the development of skin fibrosis. ET-1 may also activate neutrophils and increase NE activity, which may be associated with fibrosis development.

Low expression of inflammatory cytokines and fibrotic markers in the gadopentetate group suggested that the skin lesions observed may not be fibrotic, and that behavioral implications should be considered as a cause. Barbering is a complex behavior, which manifests as plucking whereby the dominant animals often chew off the hair of those lower in rank, resulting in hair and whisker loss [54]. Although physiological stress may also lead to behavior changes, this is rarely seen in the Slc:ddY mouse strain. Moreover, the hair bulb was found to be damaged, making barbering less likely to be the cause. Although perinatal injection of GBCAs may affect the behavior of offspring [55], there has been no evidence that Gd deposition may negatively affect brain function when used on adult subjects [56, 57]. Unfortunately, the behavioral changes that may occur as a rare side effect of GBCAs retention in the brain cannot simply be dismissed by our study.

There were several limitations in our study. We did not analyze whether there was fibrosis in other organs to confirm the cause of increased NE activity in the blood serum. Sample collection time points were based on the initial development of skin lesions, not the final injection of GBCAs. Because the development of skin lesions was not simultaneous, the mRNA results were affected by individual variability. Further studies using free forms of Gd, such as GdCl_3_, may be beneficial to determine if Gd ions may case similar effect to the skin of the renal failure mouse model.

In conclusion, although mice injected with gadodiamide developed more severe lesions, both GBCAs affect the fibrotic markers similarly. NE may play a role in the development of fibrosis, particularly in the skin, linked to the administration of GBCAs in the renal failure mouse model.
